# Renal-AI: A Deep Learning Platform for Multi-Scale Detection of Renal Ultrastructural Features in Electron Microscopy Images

**DOI:** 10.3390/diagnostics16020264

**Published:** 2026-01-14

**Authors:** Leena Nezamuldeen, Walaa Mal, Reem A. Al Zahrani, Sahar Jambi, M. Saleet Jafri

**Affiliations:** 1 The Vaccines and Immunotherapy Unit, King Fahd Medical Research Center, King Abdulaziz University, Jeddah 22254, Saudi Arabia; 2 Faculty of Applied Medical Sciences, Department of Medical Laboratory Sciences, King Abdulaziz University, Jeddah 22254, Saudi Arabia; wamal@kau.edu.sa; 3 Faculty of Medicine, Department of Pathology, King Abdulaziz University, Jeddah 22254, Saudi Arabia; raalzahrani1@kau.edu.sa; 4 Faculty of Computing and Information Technology, Department of Information Systems, King Abdulaziz University, Jeddah 22254, Saudi Arabia; shjambi@kau.edu.sa; 5 School of Systems Biology, George Mason University, Fairfax, VA 22030, USA; sjafri@gmu.edu; 6 Center for Biomedical Engineering and Technology, School of Medicine, University of Maryland, Baltimore, MD 21201, USA

**Keywords:** YOLOv8-OBB, deep learning, renal pathology, electron microscopy, object detection

## Abstract

**Background/Objectives**: Transmission electron microscopy (TEM) is an essential tool for diagnosing renal diseases. It produces high-resolution visualization of glomerular and mesangial ultrastructural features. However, manual interpretation of TEM images is labor-intensive and prone to interobserver variability. In this study, we introduced and evaluated deep learning architectures based on YOLOv8-OBB for automated detection of six ultrastructural features in kidney biopsy TEM images: glomerular basement membrane, mesangial folds, mesangial deposits, normal podocytes, podocytopathy, and subepithelial deposits. **Methods**: Building on our previous work, we propose a modified YOLOv8-OBB architecture that incorporates three major refinements: a grayscale input channel, a high-resolution P2 feature pyramid with refinement blocks (FPRbl), and a four-branch oriented detection head designed to detect small-to-large structures at multiple image scales (feature-map strides of 4, 8, 16, and 32 pixels). We compared two pretrained variants: our previous YOLOv8-OBB model developed with a grayscale input channel (GSch) and four additional feature-extraction layers (4FExL) (Pretrained + GSch + 4FExL) and the newly developed (Pretrained + FPRbl). **Results**: Quantitative assessment showed that our previously developed model (Pretrained + GSch + 4FExL) achieved an F1-score of 0.93 and mAP@0.5 of 0.953, while the (Pretrained + FPRbl) model developed in this study achieved an F1-score of 0.92 and mAP@0.5 of 0.941, demonstrating strong and clinically meaningful performance for both approaches. Qualitative assessment based on expert visual inspection of predicted bounding boxes revealed complementary strengths: (Pretrained + GSch + 4FExL) exhibited higher recall for subtle or infrequent findings, whereas (Pretrained + FPRbl) produced cleaner bounding boxes with higher-confidence predictions. **Conclusions**: This study presents how targeted architectural refinements in YOLOv8-OBB can enhance the detection of small, low-contrast, and variably oriented ultrastructural features in renal TEM images. Evaluating these refinements and translating them into a web-based platform (Renal-AI) showed the clinical applicability of deep learning-based tools for improving diagnostic efficiency and reducing interpretive variability in kidney pathology.

## 1. Introduction

Chronic kidney diseases (CKD) are widespread, affecting more than 10% of the general population throughout the globe [1]. In KSA, there are currently 1940 patients on dialysis registered in the waiting list for renal transplant, over two million cases of renal disease, and thousands of associated fatalities [2]. The lack of a national registry for kidney diseases delays the optimum resource allocation for prevention, diagnosis, and management of renal diseases. Recently, artificial intelligence (AI) has been engaging in the medical field. Developing diagnostic models using AI will improve diagnostic accuracy and save the pathologists’ time and effort. Examining renal biopsies by pathologists is essential in the diagnosis of renal diseases. The comprehensive assessment of renal biopsy requires collaborative findings of light microscopy, fluorescence microscopy, and ultrastructural evaluation by transmission electron microscopy (TEM) [3,4]. Interobserver variability among the pathologists can contribute to the findings and the turnaround time for nephrologists [5,6].

Using computer vision and AI to detect disease-specific features in medical images is an important technological development in the field of pathology, producing robust computational models for disease classification tasks. A progression in the application of AI in the field of nephropathology has been noted such as the identification and segmentation of renal structures and the evaluation of their implication in the prognosis and diagnosis of certain kidney diseases [7]. Recent literature highlights the use of AI methods such as deep learning (DL) to analyze whole slide images of renal biopsies. These approaches help find and measure histopathological features such as interstitial fibrosis, tubular atrophy, and glomerulosclerosis. For example, U-Net and R-CNN DL algorithms have demonstrated effectiveness in recognizing abnormal tubules, achieving a true positive rate of 84%. Furthermore, other DL models have been employed to diagnose specific kidney diseases, such as lupus nephritis. The ResNeXt-101 DL model, for instance, has been used to identify glomerular lesions in lupus nephritis biopsies with high accuracy, achieving an Area Under the Curve (AUC) of 0.98–0.99 [7,8,9]. In whole-slide image analysis, attention mechanisms and transformer-based architecture have been increasingly adopted for detection, segmentation, and prediction tasks [10,11,12].

The utilization of DL or attention mechanisms and transformer-based architecture in the examination of TEM images for renal biopsies remains less explored than whole-slide light microscopy [13,14,15]. The development of DL models for TEM images greatly relies on CNN-based classifiers for disease-level or deposit-level tasks [8]. More recently, AI systems for renal TEM analysis have begun to highlight the potential of transformer/attention-based models for quantitative ultrastructural assessment [16]. However, in a recent investigation conducted by [17], the researchers employed the Google Cloud AutoML model to train their MedKidneyEM-v1 classifier. The researchers focused their attention on five specific kidney diseases, namely amyloidosis, membranous nephropathy, membranoproliferative glomerulonephritis, thin basement membrane disease, and diabetic glomerulosclerosis, and a control group. They utilized 607 TEM images and labeled them according to the medical condition characteristics they present. The first model they created was designed to classify both normal conditions and renal disorders, achieving an average area under the curve (AUC) of 0.841, while the second model was specifically for classifying kidney diseases and achieved an AUC of 0.909. Another research investigation focused on the identification and analysis of electron-dense granules observed in TEM images. The TEM images that include glomeruli and electron-dense granules are considered and labelled positive, and otherwise negative. In their method they tried different machine learning approaches, such as the support vector machine (SVM) and artificial neural network (ANN) methods, to improve the accuracy of their classifier. They used the ResNet50 pretrained model, the ResNet50 + SVM model, and the ResNet50 + SVM + ANN model, the last of which showed a better F1-score of 0.88 than the first two models [18]. The third study aimed to identify electron-dense deposits in renal TEM pictures of individuals diagnosed with lupus nephritis, IgA nephropathy, and membranous nephropathy. Their approach relies on breaking down original images into several small images, focusing on the electron-dense immune deposits and labeling them as positive if they are present and negative otherwise. This investigation used several pre-trained models, including VGG16, VGG19, InceptionResNetV2, ResNet50, NASNet, and Inception-v3. The VGG19 model enhanced with several convolutional layers demonstrated higher accuracy during model validation. The F1-scores for the absence and presence of deposits were 0.76 and 0.79, respectively [19].

Our previous work [20] differs from the prior studies [17,18,19] and focuses on the identification of six features within electron microscopy images of renal biopsies. The six features are: GBM (refers to normal Glomerular Basement Membrane) found in normal kidney samples, mesangial folds (refer to the folds that found in matrix that provide support to the glomerular capillary loops), mesangial deposits (refer to deposits found within mesangial matrix), normal podocytes with intact foot process (refer to the visceral epithelial cells that is in close relation to the surface of the GBM) also found in normal kidney samples, podocytopathy (refer to an injury to the podocytes in the form of podocyte foot process effacement, detachment and microvillous transformation), and subepithelial deposits (refer to deposits located between the GBM and the visceral epithelial cell layer) [21]. The decision to incorporate two normal features into the dataset was intentional to provide the extent and progression of kidney diseases which is crucial in nephropathological assessment. For example, in some images, both normal and flattened podocytes were observed. This association offers insight into the heterogeneity of disease manifestation within a single sample. Additionally, in certain cases, GBM appeared alongside flattened podocytes. This combination suggests that disease progression may affect different kidney structures at varying rates.

We developed YOLOv8-OBB-based DL models to detect six ultrastructural features in kidney biopsy TEM images, leveraging oriented bounding boxes to accurately identify structures appearing at varying orientations. Two architectural modifications, a grayscale input channel (GSch) and four additional feature-extraction layers (4FExL), were introduced to enhance detection performance. Among four evaluated models, including the baseline (YOLOv8-OBB), (YOLOv8-OBB + GSch), (YOLOv8-OBB + 4FExL), and the combined (YOLOv8 + GSch + 4FExL) variant, the combined variant achieved the best performance (F1-score = 0.93, mAP@0.5 = 0.953). As all models demonstrated comparable performance, an ensemble prediction approach was developed. This work was presented at the 37th European Society of Pathology Congress [20]. An innovative software application (Renal-AI) was developed to automatically detect the six normal and pathological features in TEM images of kidney tissue, aiming to streamline the diagnostic process for pathologists, significantly reducing the time required to assess samples and deliver comprehensive results to nephrologists.

In this paper, we extend our previous work by introducing a revised variant of the YOLOv8-OBB architecture designed to address persistent challenges in ultrastructural renal pathology, particularly the reliable detection of small, low-contrast, and variably oriented structures in TEM images. The proposed model incorporates three major architectural refinements: (i) we preserve the grayscale input channel to better align with TEM images; (ii) The addition of a high-resolution feature pyramid map layer with refinement blocks (FPRbl) across the original pyramid map layers in the head/neck of the YOLO model to enhance multi-scale representation; (iii) A fully reconstructed four-branch OBB detection head operating at multiple image scales (strides of 4, 8, 16, and 32 pixels) to capture both small and large ultrastructural features. These modifications deepen the feature extraction process and increase sensitivity to small and low-contrast structures, such as normal podocytes, one of the common challenges in ultrastructural pathology. This study introduces how targeted architectural refinements in YOLOv8-OBB can improve the detection of small and low-contrast ultrastructural features in renal TEM images, to advance the reliability and clinical applicability of automated kidney pathology analysis.

## 2. Materials and Methods

The method comprises four phases: (1) Image collection and preprocessing, involving resolution modification and dimension standardization; (2) Image annotation and labeling, adding bounding boxes for six features; (3) Data augmentation, using a two-step strategy of grayscale conversion and brightness adjustment to enhance image quality and feature visibility; and (4) Model architecture and training, which involves training the dataset and optimizing parameters to build the final model.

### 2.1. Image Collection and Preprocessing

An archive of 607 de-identified with no patient data of electron microscope images of renal biopsies from KAUH Pathology Department (2006–2010) was obtained, plus four additional images for testing. The TIFF format black images were processed using ImageJ V.1.54p software (freely available) (URL: https://imagej.net/ij/index.html, accessed on 22 April 2023) to optimize brightness and contrast, resize to 1024 × 1024, and save as JPG files to improved visibility of the archived images.

### 2.2. Image Annotation and Labeling

Label Studio, (URL: https://labelstud.io, accessed on 3 June 2023) a free and open-source data labeling platform for academic and research use, was utilized to annotate the images with bounding boxes. This annotation process involves assigning precise coordinates to the corners of boxes drawn around objects of interest within each image. For rotated structures such as GBM, the annotation includes coordinates for all four corners (x1, y1, x2, y2, x3, y3, x4, y4) along with the corresponding class index, allowing for accurate representation of object orientation. The dataset encompasses six distinct classes: GBM, mesangial folds, mesangial deposits, normal podocytes, podocytopathy, and subepithelial deposits, thereby expanding the diversity of annotated renal structures in the electron microscope images.

**Figure 1:**
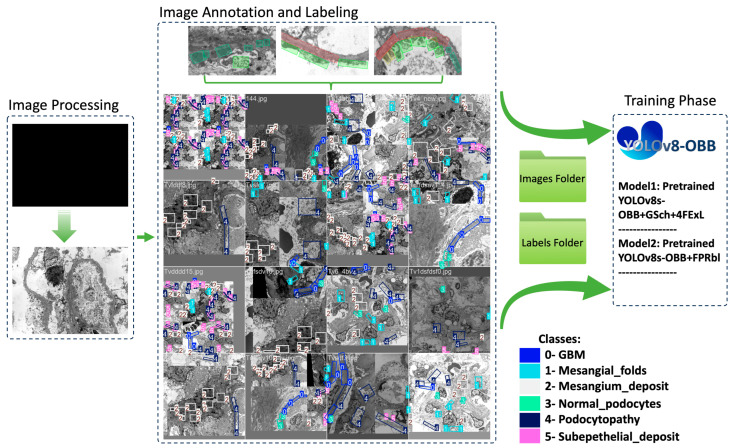
Schematic overview of the proposed YOLOv8-OBB based workflow for renal ultrastructural feature detection in TEM images, including image preprocessing using ImageJ, annotation of six ultrastructural features utilizing Label Studio web application, data augmentation to increase the dataset size, and training of modified YOLOv8-OBB architecture with grayscale input (GSch), additional feature-extraction layers (4FExL), and feature pyramid refinement blocks (FPRbl).

After the annotations were made, the annotated dataset was exported in YOLOv8-OBB format, consisting of two main folders and a YAML file. The first folder contains images split into training (73.5%) and validation (26.5%) datasets, while the second folder holds corresponding text files with bounding box coordinates. The YAML file serves as metadata, providing file paths and class lists (Figure 1).

**Figure 2:**
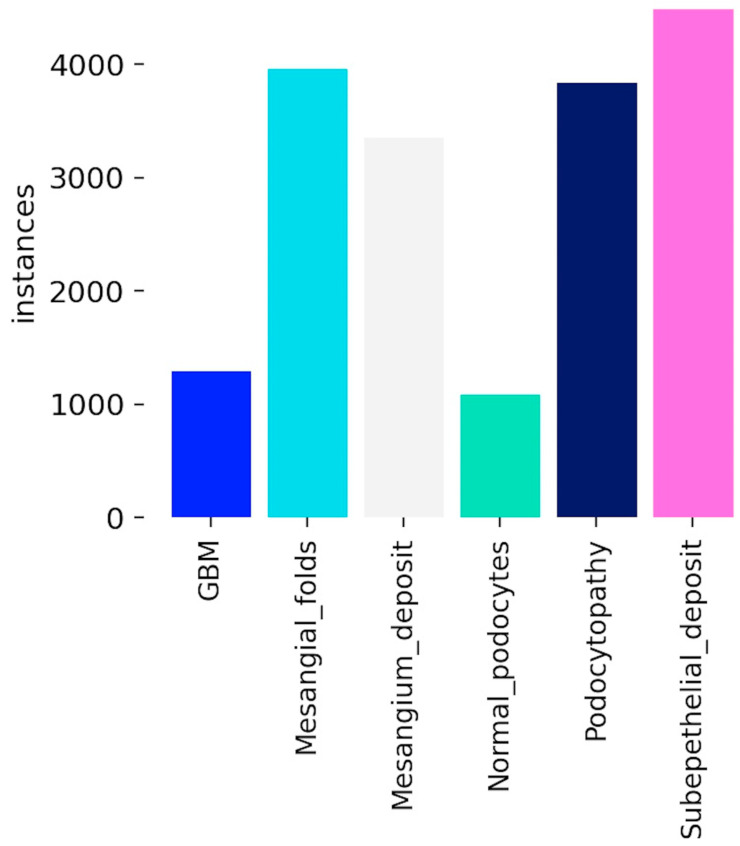
Distribution of annotated instances across different histological categories in the dataset. Categories include GBM, mesangial folds, mesangial deposits, normal podocytes, podocytopathy, and subepithelial deposits. The variation in counts highlights the class imbalance, with subepithelial deposits having the highest frequency and GBM the lowest.

Because of the association of some structural features, such as the correlation between subepithelial deposits and podocytopathy, the distribution of bounding boxes (instances) that represent the annotated features for each of the six classes is unevenly spread across the images (Figure 2). Furthermore, GBM and normal podocytes are normal features that are found rarely in images for pathological nephrology conditions.

### 2.3. Image Augmentation

To enhance the diversity and size of our electron microscope image dataset, we applied two augmentation techniques: grayscaling and brightness adjustments. These augmentations were implemented using the OpenCV library in Python [22]. Each color image was converted to grayscale using cvtColor function to emphasize structural details and reduce dimensionality. To expand variations in imaging conditions, we adjusted the brightness of the images using convertScaleAbs function with a contrast factor (alpha) of 1.5 and a brightness factor (beta) of 50. This is the equation that represents the function of convertScaleAbs:

(1) dst(I)=saturate cast uchar(|src(I)×α+β|)

where dst(I) is the output pixel value, src(I) is the input pixel value, alpha is the scaling factor that controls contrast, beta is the offset value that controls brightness, and saturate_cast_uchar ensures the result is within the range of an unsigned 8-bit integer, preventing overflow or underflow. During the augmentation process, special care was taken to ensure that the location of bounding boxes around objects of interest were preserved.

### 2.4. Model Architecture and Training

In this study, we applied three architectural refinements to the original YOLOv8-OBB model: (i) modifying the input stem to accept a single grayscale channel, (ii) adding a high-resolution P2 feature map and refinement blocks to the original P3–P5 pyramid levels, and (iii) replacing the detection head with a four-branch OBB head operating at strides of (4, 8, 16, 32 pixels). These changes improve multi-scale feature extraction and enhance detection of small and low-contrast ultrastructural features.

#### Yolo and YOLOv8-OBB Algorithms Overview

YOLO (You Only Look Once) developed by Ultralytics [23] is a real-time object detection algorithm that predicts multiple objects in an image or video frame in a single pass with single-stage architecture of backbone, neck, and head. It divides the input image into a grid, with each cell predicting bounding boxes, class probabilities, and a confidence score:

Confidence Score = Pr(object) × IoU(2)

Here, “Pr(object)” refers to the probability of an object being present within the box, and “IoU” represents the Intersection over Union that measures the overlap between predicted and ground truth boxes. In 2023, the YOLOv8 model was developed [24]. It is pre-trained on the Microsoft COCO dataset and Pascal VOC (visual object classes). YOLOv8-OBB variant capable of detecting rotated structure and is pretrained on additional dataset named DOTA, (Dataset for Object deTection in Aerial images) dataset (Ultralytics. (2024). YOLOv8-OBB. https://github.com/ultralytics/ultralytics, accessed on 10 June 2023). The YOLOv8-OBB model is available in various sizes, and in this study, the YOLOv8s-OBB model small was used. The architecture of the model consists of:

- **Backbone:** CBS, C2f, and SPPF modules for hierarchical feature extraction
- **Neck:** PANet and C2f modules for multi-scale feature fusion
- **Head:** OBB detection modules that output rotated bounding boxes through dynamic anchor assignment and specialized loss components

### 2.5. The Proposed Modified Architecture

Unlike standard YOLOv8-OBB architectures designed for natural image detection, the proposed model introduces task-specific modifications to the feature pyramid and detection head to address the unique challenges of renal TEM imaging, including small feature size, low contrast, and variable orientation. The original YOLOv8 architecture employs a hierarchical feature pyramid in which maps at different spatial resolutions (denoted as *P*-levels) are used to detect objects of varying sizes. Each *P*-level corresponds to a specific down sampling factor, known as the stride, which determines the resolution of the feature map relative to the input image. In the standard YOLOv8-OBB model, prediction heads operate on three pyramid levels P3, P4, and P5 with strides of 8, 16, and 32, respectively. These levels enable the model to detect small, medium, and large structures in an image. In the proposed modified architecture, a fourth high-resolution feature map (P2) was introduced to enhance the model’s sensitivity to fine-scale structures that are characteristic of renal ultrastructure. The P2 feature map is generated by up sampling the original P3 output and refining it using a series of convolution–batch normalization–ReLU layers.

**Figure 3:**
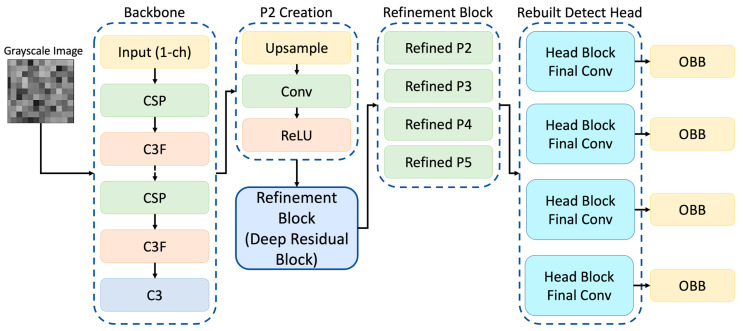
Overview of the modified YOLOv8s-OBB model used for renal TEM feature detection. The network includes a grayscale input stem (1-channel), a standard backbone for hierarchical feature extraction, and the introduction of a new P2 feature layer generated via upsampling and convolution. Refinement blocks (deep residual modules) are applied to all pyramid levels (P2–P5) before prediction. The detection head is rebuilt into four parallel branches, each ending in an OBB output for multi-scale feature detection.

Refinement blocks were also applied to the original P3, P4, and P5 levels to improve feature consistency across the pyramid (Figure 3). As a result, the detection head now operates on four pyramid levels with strides (4, 8, 16, 32) corresponding to feature maps at progressively reduced spatial resolutions:

- **P2 (stride 4):** very small structures
- **P3 (stride 8):** small structures
- **P4 (stride 16):** medium-sized structures
- **P5 (stride 32):** large structures

This expanded pyramid improves multi-scale representation and enhances detection of subtle or low-contrast features such as podocyte foot processes, basement membrane contours, and other cellular details. To align the model with the imaging modality, the input stem of the network was modified to accept single-channel grayscale TEM images by converting the initial convolutional layer from three channels to one. Additionally, the original YOLOv8-OBB detection head was replaced with a newly constructed four-branch OBB head, in which each branch consists of multiple convolutional refinement layers followed by an output layer predicting the eight corner coordinates and class label for each object.

### 2.6. Training Environment and Implementation Details

Models training were implemented in Python (Version 3.10). Experiments were conducted using PyTorch (version 2.7) on a Supercomputer (AZIZ) equipped with an NVIDIA GPU, Santa Clara, USA (NVIDIA A100 with 40 GB VRAM). Image preprocessing and augmentation were implemented using OpenCV and NumPy libraries. All models were trained with an input resolution of 1024 × 1024 pixels using stochastic gradient descent (SGD) for 300 epochs, with a learning rate of 0.01 and batch size of 16.

### 2.7. Model Evaluation

When evaluating YOLOv8 object-detection models, key metrics include precision, recall, F1-score, and mean average precision (mAP), with mAP being the main benchmark. Precision measures the ratio of true positives to all positive detections (true positives plus false positives).

(3) $$\mathrm{Precision}=\frac{True\;positive}{True\;positive+False\;positive}$$

Recall is the ratio of true positives to all actual positives (true positives plus false negatives).

(4) $$\mathrm{Recall}=\frac{True\;posistive}{True\;positive+False\;negative}$$

F1-Score is a measure of a model’s performance’s accuracy of object detection tasks. It depends on the precision and recall of the model training.

(5) $$\mathrm{F1-Score}=2\times \frac{Precision\times Recall}{Precision+Recall}$$

Average Precision (AP) measures the area under the precision-recall curve, evaluating model performance across multiple intersection over union (IoU) thresholds from 0.5 to 0.95. The IoU score, calculated as the intersection over union of predicted and ground truth boxes, helps assess detection accuracy at different standards. AP is computed for each class and averaged to produce mean Average Precision (mAP), a key metric for overall object detection performance. In this study, mAP at an IoU threshold 0.5 (mAP@0.5) was used, which considers a detection correct if the IoU is at least 0.5, a widely used matrix in the field of object detection tasks.

(6) $$mAP=\frac{1}{N}\sum_{1}^{N}AP$$

## 3. Results and Discussion

The original dataset consisted of 607 images, each with dimensions of 1024 × 1024. After applying the image augmentation, the dataset was expanded to include 1652 augmented images. These images were then used to train the DL models. The number of images in the training dataset was 1376 (83% of the total images) and the number of images in the validation dataset was 276 (17% of the total images).

### 3.1. Experimenting the Proposed Model

The performance of the previously developed pretrained YOLOv8-OBB models [20] were compared with the newly proposed model in this paper (Pretrained + FPRbl) to evaluate the impact of the architectural modifications (Table 1).

In our previous study, the Pretrained YOLOv8s-OBB + GSch + 4FExL model achieved the highest overall performance, with an F1-score of 0.93 and an mAP@0.5 of 0.953, surpassing all other evaluated architectures. Therefore, in the present study, we focused our comparison on this top-performing model and the newly proposed (Pretrained YOLOv8s-OBB + FPRbl) architecture, rather than including the remaining previously assessed models. This targeted comparison was intended to determine whether the newly developed model could compete with, or potentially surpass, the best-performing configuration from our earlier work.

**Figure 4:**
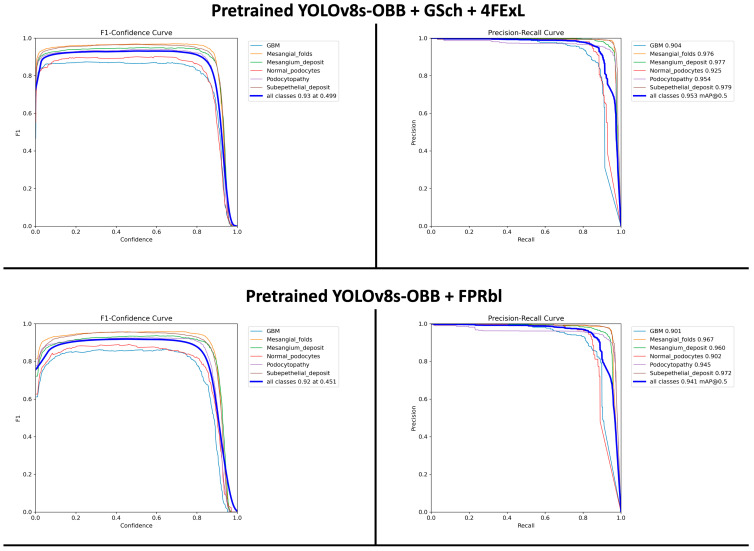
F1-confidence and precision–recall curves for the two YOLOv8s-OBB model configurations. The top row shows results for the Pretrained + GSch + 4FExL model, and the bottom row shows results for the Pretrained + FPRbl model. Per-class curves are displayed for all six ultrastructural renal features, along with the aggregated performance across classes. The Pretrained + GSch + 4FExL model achieved an optimal F1-score of 0.93 at a confidence threshold of 0.499 and an mAP@0.5 of 0.953, whereas the Pretrained + FPRbl model achieved an optimal F1-score of 0.92 at 0.451 and an mAP@0.5 of 0.941.

The F1-scores and mAP@0.5 values for both models, along with their relative performance differences, are illustrated in Figure 4. The results (Figure 4) demonstrate the effect of these structural changes on YOLOv8s-OBB detection accuracy. Previously, the Pretrained + GSch + 4FExL model achieved an F1-score of 0.93 at a confidence threshold of 0.499 and an mAP@0.5 of 0.953, outperforming the newly proposed configuration. The (Pretrained + FPRbl) model produced a closely comparable F1-score of 0.92 at a threshold of 0.451 and an mAP@0.5 of 0.941. These findings indicate that although the previously developed model shows slightly stronger performance, both architectures demonstrate robust and accurate detection at an IoU threshold of 0.5.

### 3.2. Models’ Detection and Visualization

**Figure 5:**
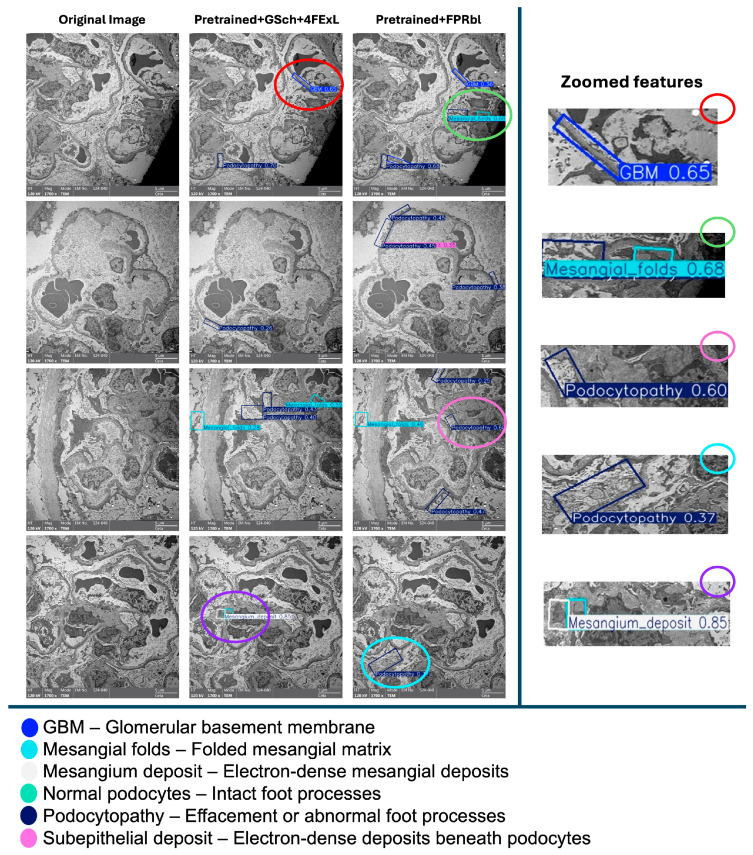
An illustration of a qualitative comparison between prediction outputs on four representative TEM images (left columns). The (Pretrained + GSch + 4FExL) model produced a greater number of detections overall, including several subtle low-contrast features, although with multiple overlapping bounding boxes and moderate confidence values. In contrast, the (Pretrained + FPRbl) model generated fewer detections but generally with higher confidence scores and less redundancy. Notably, the (Pretrained + GSch + 4FExL) model correctly identified a mesangial deposit (confidence 0.85), whereas the (Pretrained + FPRbl) model missed this structure and instead produced an unrelated podocytopathy detection (confidence 0.37). These observations align with the quantitative metrics (Table 1), indicating that the first model favors recall, while the second favors precision. Zoomed-in panels (right) highlight representative detections of the glomerular basement membrane (GBM), mesangial folds, and podocytopathy to aid visual interpretation. Scale bar = 5 µm (main images).

On electron microscopy, normal podocytes are characterized by intact, regularly spaced interdigitating foot processes with preserved slit diaphragms, whereas podocytopathy is defined by foot process effacement, broadening or fusion of foot processes, and disruption of normal slit diaphragm architecture, consistent with established renal ultrastructural diagnostic criteria. A comparative visual analysis was conducted to evaluate the outputs of (Pretrained + GSch + 4FExL) and (Pretrained + FPRbl) models against the original image. Each model demonstrates distinct detection patterns with varying confidence levels, while the ensemble method integrates these predictions, potentially enhancing accuracy through combined outputs. The left images present the original image without any detections, serving as a baseline reference. The remaining images showcase detection results from the two models, each highlighting different structural features. Zoomed-in panels (right) provide magnified views of representative regions, emphasizing detections of the glomerular basement membrane (GBM), mesangial folds, and podocytopathy to facilitate visual interpretation. The color-coded legend maps each bounding box color to the corresponding detected feature, offering clarity on the classifications displayed in the (Figure 5).

Visual comparison of prediction outputs (Figure 5) demonstrates consistent differences between the two YOLOv8-OBB configurations. Across representative TEM images, the (Pretrained + GSch + 4FExL) model generated a larger number of bounding boxes, often identifying subtle or low-contrast structures such as mesangial folds and podocytopathy that were not captured by the (Pretrained + FPRbl) model. In several cases, the (Pretrained + FPRbl) variant produced fewer detections overall but with higher individual confidence scores and cleaner, less overlapping predictions. Specifically, in the first comparison image, the (Pretrained + GSch + 4FExL) model detected GBM and podocytopathy with higher confidence, whereas the (Pretrained + FPRbl) model identified GBM with lower confidence but additionally detected a mesangial fold. In following images, both models detected mesangial and podocyte regions, although the (Pretrained + FPRbl) predictions tended to be sharper with greater confidence.

The most notable difference appeared in the final image, where the (Pretrained + GSch + 4FExL) model correctly detected a mesangial deposit at high confidence (0.85), whereas the (Pretrained + FPRbl) model missed this feature and returned a lower confidence podocytopathy prediction (0.37). Overall, the (Pretrained + GSch + 4FExL) model demonstrated improved recall for rare or subtle abnormalities, while the (Pretrained + FPRbl) model favored precision, producing fewer but more confident detections across the evaluated test images.

### 3.3. Web Application for Renal-AI

We developed Renal-AI, a web-based application designed to detect and analyze ultrastructural features in renal electron microscopy images using the ensemble method from our previous study.

**Figure 6:**
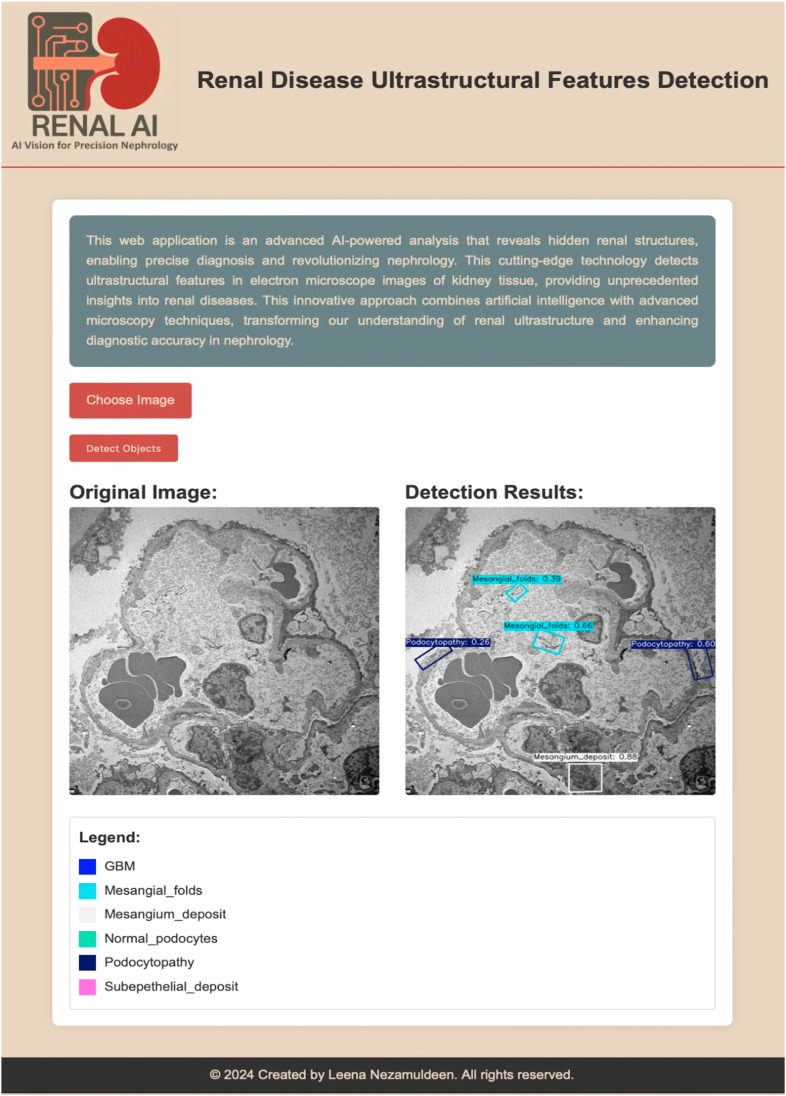
AI-powered renal disease ultrastructural feature detection interface. The application enables precise detection of renal structures in electron microscopy images of kidney tissue. The left panel shows the original input image, while the right panel displays detection results with annotated regions and confidence scores. The legend indicates the detected features, including glomerular basement membrane (GBM), mesangial folds, mesangial deposits, normal podocytes, podocytopathy, and subepithelial deposits. Scale bar = 5 µm.

The platform offers an intuitive interface where users upload EM images, and the system automatically identifies and annotates key renal structures such as glomerular basement membranes, mesangial alterations, and podocytes using labeled bounding boxes and confidence scores (Figure 6). In this paper, the ensemble framework is expanded by incorporating the newly developed (Pretrained + FPRbl) model alongside our prior models (Pretrained YOLOv8s-OBB), (Pretrained YOLOv8s-OBB + 4FExL), (Pretrained YOLOv8s-OBB + GSch), and (Pretrained YOLOv8s-OBB + GSch + 4FExL). Integrating both architectures enhances prediction robustness, reduces missed detections, and improves consistency across low-contrast and heterogeneous ultrastructural regions. Renal-AI provides a scalable, accessible solution that advances precision nephrology by streamlining the identification of critical renal features and supporting improved diagnostic workflows.

This study aimed to enhance the automated detection of renal ultrastructural features in TEM images through introducing certain architectural changes to the YOLOv8-OBB framework. We assessed whether improvements in multi-scale feature representation and oriented detection heads may enhance sensitivity and localization accuracy for small, low-contrast, and variable orientated renal structures. Our findings indicate that both evaluated architectures achieved robust and clinically significant performance, demonstrating complementary strengths in recall and precision, so advancing the study’s objective of enhancing reliable and interpretable AI-assisted renal pathology analysis.

The diagnosis of renal diseases is a complex procedure that mainly relies on the analysis of kidney biopsy images, including TEM, which usually requires professional renal pathologists and subjective visual evaluation. Currently, a limited number of studies have utilized deep learning methodologies on TEM images to investigate renal diseases. Previous studies predominantly focused on disease-level classification tasks, using ultrastructural imaging features to distinguish types of renal disease rather than identifying individual ultrastructural features [17,18,19]. In contrast, our research presents innovative architectural alterations to YOLOv8-OBB models for the object-level detection of renal ultrastructural characteristics in TEM images. Instead of immediately identifying disease entities, our methodology emphasizes the detection of both normal and pathological ultrastructural characteristics, regardless of the illness kind. This feature-oriented technique allows for more detailed and flexible analysis, offering improved assistance to nephropathologists by enabling the evaluation of disease severity and underlying pathology through the clear identification of ultrastructural abnormalities.

Our study evaluated two YOLOv8-OBB pretrained based deep learning architectures designed for automated detection of six ultrastructural features in TEM kidney biopsy images. Choosing pretrained version was to leverage knowledge from large-scale datasets through pretraining. This significantly enhances the model’s capacity to recognize complex pathological structures in kidney biopsy images. The primary objective was to determine whether targeted architectural refinements to the feature pyramid and detection head would improve performance relative to our previously optimized model. Quantitatively, the Pretrained + GSch + 4FExL model demonstrated slightly stronger performance, achieving an F1-score of 0.93 and mAP@0.5 of 0.953, compared to an F1-score of 0.92 and mAP@0.5 of 0.941 for the newly proposed (Pretrained + FPRbl) model. Although this difference is minor, both models achieved accuracy levels considered clinically meaningful for object detection tasks in medical imaging. Our mAP and F1-score for both models are within the high-performance range reported by recent deep learning applications in renal pathology and computational nephropathology [7,8,25,26]. Comparison of qualitative prediction outputs showed differing detection characteristics between the two models. The (Pretrained + GSch + 4FExL) model generated a larger number of bounding boxes and demonstrated higher recall, particularly for subtle and low-contrast features such as podocytopathy, mesangial folds, and GBM. However, this sensitivity was sometimes accompanied by redundant or overlapping predictions. In contrast, the (Pretrained + FPRbl) model produced fewer detections with higher confidence scores and reduced spatial noise, reflecting improved precision. A notable example was observed in cases involving rare lesions such as mesangial deposits: the (Pretrained + GSch + 4FExL) model detected a true positive with high confidence, whereas the (Pretrained + FPRbl) model missed this feature and returned a lower-confidence misclassification. These findings illustrate a typical precision–recall trade-off in object detection and suggest that the two architectures capture complementary information.

From a computational nephropathology perspective, the ability to balance recall and precision has practical implications. High recall reduces the likelihood of missing clinically relevant structures during initial screening, while high precision minimizes false positives and visual clutter, improving interpretability for pathologists. The complementary strengths observed between the two models support the use of an ensemble method, in which predictions from multiple architectures are integrated to achieve more robust performance. This approach has already demonstrated benefits in our prior work and is further strengthened by the inclusion of the newly proposed (Pretrained + FPRbl) model. TEM images of renal biopsies present inherent challenges, including low contrast, heterogeneous tissue morphology, and limited availability of annotated datasets [18,26]. The introduction of a P2 high-resolution feature map and refinement blocks across the P3–P5 pyramid levels was therefore well justified. The use of oriented bounding boxes further improves localization of oblique or branching structures, overcoming limitations of axis-aligned detection common in natural image datasets. Nonetheless, domain-specific ambiguities remain; visual similarity between normal and pathological podocyte features, heterogeneity within mesangial regions, and variability in sample preparation can confound model discrimination.

### 3.4. Limitations of the Study

While pretrained models and ensemble approach significantly improved feature detection accuracy and reliability, especially in complex regions, limitations persist and need to be acknowledged. This study used transmission electron microscopy images obtained from a single institution, which could introduce center-specific bias linked with local diagnostic methods, disease prevalence, and imaging protocols. Additionally, the dataset demonstrated class imbalance, with specific ultrastructural features, such as subepithelial deposits, appearing more frequently than others. This variation could bias the models towards the detection of more often represented components. Moreover, several factors may affect model performance. Differences in image quality, including contrast, noise levels, section thickness, and sample preparation, can change the ultrastructural features appearance. Disease heterogeneity also presents a challenge, as the same ultrastructural feature may exhibit differently across disease types or stages. Another important limitation is the generalizability. The rare disease patterns in rare kidney diseases are not present in Saudi Arabia but are prevalent in other continents where incidence rates and disease spectra differ due to genetic, environmental, and demographic factors. To ensure broader applicability and diagnostic accuracy, it is essential to include the ultrastructural features that define these additional diseases in our models, drawing on multi-regional datasets and collaborative efforts with international centers.

Addressing these limitations will require the integration of multi-modal data, as well as collaborative efforts between computational researchers and clinical experts. This approach will help capture the full spectrum of global renal pathology, enabling AI systems to recognize and differentiate rare conditions that may otherwise go undetected in local datasets, and ultimately support more equitable and comprehensive kidney disease diagnosis worldwide. Future work would focus on expanding datasets, refining model interpretability, and validating these tools in clinical settings to ensure robust, clinically meaningful AI-assisted renal pathology analysis that enhances diagnostic precision while leveraging the expertise of human pathologists.

## 4. Conclusions

In this study, we advanced automated ultrastructural analysis of renal electron microscopy images by developing and evaluating a modified YOLOv8-OBB architecture tailored for the detection of six clinically relevant renal features. Building upon our previous work, we introduced a refined model incorporating a high-resolution P2 feature map, feature pyramid refinement blocks, and a redesigned four-branch OBB detection head. Comparative analysis demonstrated that while the previously developed Pretrained + GSch + 4FExL model achieved the highest overall performance (F1-score 0.93; mAP@0.5 0.953), the newly proposed Pretrained + FPRbl model produced competitive results (F1-score 0.92; mAP@0.5 0.941) and offered improved precision with cleaner, higher-confidence detections. Qualitative assessments further indicated that the two models capture complementary aspects of renal ultrastructure, with the former model being sensitive to thin features and the latter model offering more localization accuracy.

These findings underscore the value of targeted architectural refinements for enhancing performance in low-contrast, heterogeneous, and small-scale biomedical imaging tasks such as TEM-based renal pathology. By integrating both models into the Renal-AI platform, we provide a practical tool that improves detection consistency, reduces interpretive variability, and supports nephropathologists in analyzing complex ultrastructural patterns. Future work will focus on expanding the dataset to include a broader spectrum of global renal diseases, improving model interpretability, and conducting multi-center validation to ensure clinical robustness. In conclusion, this research demonstrates that deep learning frameworks hold strong potential to transform renal EM image interpretation and to advance precision diagnostics in nephropathology.

## Figures and Tables

**Table 1:** Comparative performance metrics for YOLOv8s-OBB models. For each model, the reported F1-score is shown at the confidence threshold that maximized the F1-score on the validation set, with the corresponding threshold value indicated. Mean average precision (mAP@0.5) denotes the average detection accuracy across all six annotated ultrastructural feature classes. FPRbl = feature pyramid refinement blocks; GSch = grayscale channel; 4FExL = four feature-extraction layers.

Model	F1-Score	mAP@0.5
Pretrained YOLOv8s-OBB	0.92 at 0.532	0.936
Pretrained YOLOv8s-OBB + GSch	0.92 at 0.560	0.935
Pretrained YOLOv8s-OBB + 4FExL	0.91 at 0.422	0.930
Pretrained YOLOv8s-OBB + GSch + 4FExL	0.93 at 0.499	0.953
Pretrained YOLOv8s-OBB + FPRbl *	0.92 at 0.451	0.941

* New proposed models.

## Data Availability

Data is not available due to the privacy of patients’ information.

## Abbreviations

The following abbreviations are used in this manuscript:

TEM	Transmission electron microscopy
CKD	Chronic kidney diseases
AI	Artificial Intelligence
DL	Deep Learning
GBM	Glomerular Basement Membrane
OBB	Oriented Bounding Boxes
AUC	Area Under the Curve
AP	Average Precision
mAP	mean Average Precision
IoU	Intersection over Union
SGD	Stochastic Gradient Descent
VOC	visual object classes
FPRbl	Feature Pyramid Refinement Blocks
GSch	Grayscale Input Channel
4FExL	Four Feature Extraction Layers
